# A social media analysis of patient-perceived complications following periacetabular osteotomy (PAO): a retrospective observational study

**DOI:** 10.1186/s12893-024-02318-1

**Published:** 2024-01-24

**Authors:** Bretton Laboret, Ryan Bialaszewski, John Gaddis, Emily Middleton, Brittany Kendall, Katie Lynch, Adina Stewart, Joel Wells

**Affiliations:** 1 University of Texas Southwestern Medical School, Dallas, TX USA; 2 University of Texas Rio Grande Valley, Edinburg, TX USA; 3 Department of Physical Therapy - School of Health Professions, University of Texas Southwestern, 6011 Harry Hines Blvd., Dallas, TX 75390-8876 USA; 4 Baylor Scott & White Outpatient Rehabilitation - Frisco, 3800 Gaylord Pkwy Ste 130, Frisco, TX 75034 USA; 5 Baylor Scott & White Outpatient Rehabilitation - Frisco, 3800 Gaylord Pkwy Ste 130, Frisco, TX 75034 USA; 6 Department of Orthopedic Surgery, Baylor Scott and White Hip Preservation Center, 5220 W University Dr Ste 220, McKinney, TX 75071 USA

**Keywords:** Periacetabular Osteotomy, Hip Dysplasia, Complications, Social media

## Abstract

**Background:**

Social media is a popular resource for patients seeking medical information and sharing experiences. Periacetabular osteotomy (PAO) is an accepted treatment for symptomatic acetabular dysplasia with a low published complication profile in specialty centers. Little is known regarding patient reporting of complications on social media following PAO. The purpose of this study was to describe the patient-perceived complications of PAO posted on social media and analyze how additional factors (postoperative timeframe, concomitant surgery) correlate with these complication posts.

**Methods:**

Facebook and Instagram were queried from 02/01/18–02/01/23; Twitter was searched over an extended range back to 02/01/11. Facebook posts (1054) were collected from the two most populated interest groups; “Periacetabular Osteotomy” and “PAO Australia.” Instagram posts (1003) and Tweets (502) were found using the same five most popular hashtags: #PAOwarrior, #periacetabularosteotomy, #periacetabularosteotomysurgery, #PAOsurgery, and #PAOrecovery. Posts were assessed for demographic data, perspective, timing (early postoperative or late postoperative), additional surgeries, type of complication, and post engagement.

**Results:**

Facebook posts (1054), Instagram posts (1003), and Tweets (502) were assessed; 13.6% of posts included a complication. The majority of complications were reported > 6 months postoperatively with excessive pain being the most common complication (57.2%), including chronic pain (41.8%), acute pain (6.7%), and nerve pain (8.8%). Bony complications (6.7%), neurologic/psychiatric complications (3.8%), swelling (1.7%), infection (1.4%), other specified complications (16.2%), and unspecified complications (10.2%) were reported. Complication posts were found to be correlated with postoperative timeframe and concomitant surgery. Post engagement decreased in complication-related posts.

**Conclusions:**

Few patients posted a perceived complication associated with PAO surgery. Of those who did, the majority reported unmanageable pain during the late postoperative period. Posts including a perceived complication were found to be positively correlated with postoperative timeframe and negatively correlated with concomitant surgery. This study found a higher pain complication rate, but a lower overall complication rate compared to prior studies. Considering the social media reported complications of PAO patients in addition to traditional outcome measures reveals which aspects of postoperative recovery are most important to patients themselves.

## Background

The advent and rapid expansion of social media networking over the past two decades has allowed patients to seek medical advice, learn about their condition, and share their experiences with others on their own terms. Patient reporting via social media has been successful with several advantages over traditional patient reported outcome measures (PROMs), including lowered costs, shortened response times, and an increased ability for healthcare providers to examine large amounts of patient data from anywhere in the world [1–9]. A recognized concern with PROMs is that patients may not answer questions with complete accuracy in a healthcare setting [10–14].

Several studies have examined the use of social media platforms to gather patient perspectives for a variety of orthopedic as well as other surgical interventions, including hip arthroscopy, hand and upper extremity surgery, breast reconstructive surgery, brain aneurysm repair, and migraine surgery [1–9]. Most of these studies focus on collecting posts in the perioperative or immediate postoperative periods, with fewer studies examining longer term complications. To our knowledge, no prior study utilizing social media to assess patient perspectives of the periacetabular osteotomy surgery has been performed to date.

The Bernese Periacetabular Osteotomy (PAO) is a successful surgical alternative to total hip arthroplasty (THA) for developmental hip dysplasia (DDH) [15–17]. Periacetabular dysplasia is the most common cause of secondary hip arthrosis, affecting upwards of 50% of patients with developmental hip disease by age 50 [15, 18]. PAO has demonstrated both short-term improvements in pain level and function as well as prolonged the requirement for joint replacement and has a low complications profile in the literature [16, 17, 19, 20]. Complications associated with the surgery can vary from manageable pain to secondary invasive procedures that may present significant changes to patient quality of life [15, 20]. The aim of this study is to use social media posts to create a more complete depiction of the patient-perceived complications of PAO, providing physicians with information to assess their current methods for mitigating complications and managing patient expectations.

We therefore sought to answer the following questions: (1) What are the patient-perceived complications of PAO posted on Facebook, Instagram, and Twitter? (2) How do additional factors (postoperative timeframe, concomitant surgery) correlate with these complication posts? (3) Do the PAO complications assessed for by physicians postoperatively align with perceived complications that patients are reporting?

## Methods

Two-thousand five hundred and fifty-nine (2559) social media posts related to PAO surgery were collected retrospectively from 02/01/2023 to 02/01/2018 from three social media platforms: Facebook, Instagram, and Twitter. One thousand fifty-four (1054) posts were collected on Facebook from the two most populated PAO-specific interest groups, “Periacetabular Osteotomy” and “PAO Australia.” One thousand three (1003) unique posts from Instagram and 502 from Twitter were collected. To be included on these two platforms, each post contained at least one of the five most popular hashtags associated with PAO: #PAOwarrior, #Periacetabularosteotomy, #periacetabularosteotomyrecovery, #PAOsurgery, and #PAOrecovery. Tweets were gathered over an extended period back to 02/01/2011.

Only posts that were related to PAO surgery and met search criteria were included in initial analysis. Of these, posts that included a complication were further analyzed as outlined below. Posts not related to PAO surgery were excluded, as were posts not in the English language.

**Fig. 1:**
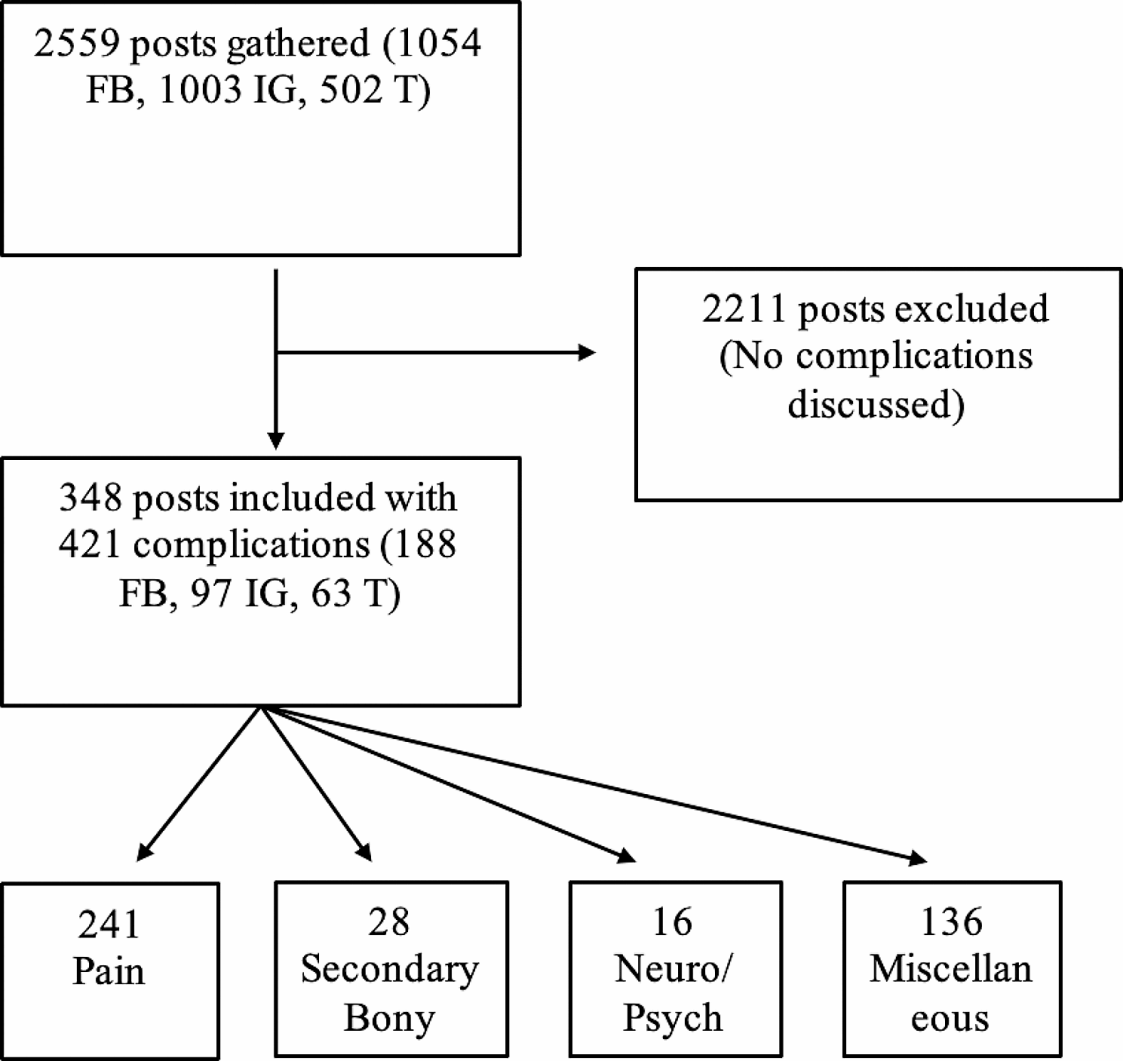
A flow diagram of the patient posts selected is shown

Selected posts were assessed and categorized on an extensive criterion: demographic data (sex, location), perspective (patient, physician, family, industry, organization), timing (early postoperative complications or late postoperative complications), post engagement (shares, comments, likes), concomitant surgeries, and perceived complications. The patient-perceived complications examined in the study are based on prior publications on postoperative complications common for PAO, and included nerve pain, anemia, infection, nonunion, syncope, constipation, chronic pain, allergic reaction, acute pain (defined as < 1 week), secondary fracture, and depression/mental health change [17, 20–22]. Additional patient-perceived complications of sleep issues, swelling, headache, POTS, bursitis, non-specified complication, and other complications were included. Figure 1 demonstrates the selection process for posts.

Average postoperative dates of posts were calculated to evaluate the overall timeframe of patients using social media for discussion of surgical complications. Measurements of post engagement (likes, shares, and comments) were collected to distinguish between the level of user interaction among patient posts on the three platforms. To ensure posts were categorized consistently and to minimize bias, each author reviewed an additional twenty posts collected from other authors, with high agreeance (93.3% inter-observer agreement when analyzing complication categorization). Data analysis was performed in Microsoft Excel using researcher created t-tests for two-variable analysis and one-way ANOVA for three-variable analysis. To ensure validity, all statistical analysis was performed independently by two authors.

## Results

A total of 1054 Facebook posts, 1003 Instagram posts, and 502 Tweets from 887 unique authors were consecutively categorized (2559 total posts). 87.0% of these were posts from patients, 6.1% from family members, and 3.6% from a professional organization. The other 6.3% of posts were from physicians, hospitals, news media, and physical therapy groups. Overall, 2193 (89.4%) posts were from females, 99 (4.0%) posts were from males, and 161 (6.6%) posts were from posters of unknown gender. Five hundred and three (78.0%) posts were from North America, 121 (18.6%) posts were from Europe, 14 (2.2%) posts were from Australia or New Zealand, and 7 (1.3%) posts were from other countries. The top fifteen individual posters on all three sites accounted for 17.7% of posts. Of the 2559 posts, 1684 (65.8%) were published postoperatively, with an average timeframe of 321 days after surgery (Median 84 days, IQR 20–275 days) for those on whom data concerning date of operation could be collected. Of the 1684 postoperative posts, 348 posts consisted of a reported complication (13.6% of total posts).

Four hundred and twenty-one (421) total complications were reported in 348 posts, including 122 complications from 97 Instagram posts, 209 complications from 188 Facebook posts, and 90 complications from 63 Tweets. Regarding gender, women were more likely to post about PAO in general, and even more likely to write a post describing a complication (89.7% vs. 94.0%, *p* < 0.00001). Facebook accounted for nearly half of all posts with a perceived complication (209 posts) and Facebook posts were more likely than Twitter or Instagram to note a complication (49.6% vs. 29.0%, *p* < 0.001485). Furthermore, posts including a complication were made an average of 348 days postoperatively, while posts without a complication were made an average of 246 days after surgery. Posts including a complication were more likely to occur later in the postoperative timeframe than posts without a complication (t(275)=-3.47, *p* < 0.0006). However, there was no significant difference between the average postoperative timeframe for complication posts between each social media site. Table 1 shows postoperative timeframes for each social media site.

**Table 1:** Postoperative timeframe of complication vs. non-complication posts

	Facebook	Instagram	Twitter	Average
Complication Post	548 days	220 days	277 days	348 days
Non-complication Post	286 days	176 days	277 days	246 days

Perceived complications were subdivided into four categories: (1) unmanageable pain (chronic, acute, nerve), (2) bony complications (non-union, fracture, progression to THA), (3) neurological complications (sleep issues, headache, POTS, mental health changes), and (4) miscellaneous/other complications (swelling, infection, allergic reaction, other specified complications, and non-specified complications). Table 2 summarizes the results for all three social media sites. The majority of patient-reported complications were related to unmanageable pain, accounting for 241 posted complications (57.2%). This includes 28 posts regarding acute pain < 1 week postoperatively (6.7% of complications, average of 3.5 days postoperatively), 176 posts regarding chronic pain > 1 week postoperatively (41.8% of complications, average of 211 days postoperatively), and 37 posts regarding nerve pain (8.8% of complications, average of 253 days postoperatively). Chronic pain was the greatest proportion of complication-related posts on all three sites (41.8% of complications). Facebook and Instagram posts accounted for 85% of reported pain complications and were equally likely to have a post referencing unmanageable pain (43.6% vs. 41.5%, *p* = 0.995145). 28 reported complications were related to secondary bone dysfunction (6.7% of complications), with 16 nonunion (3.8% of complications), 10 secondary fractures (2.4% of complications), and 2 progressions requiring a THA (0.5% of complications). 42 perceived complications were classified as ‘non-specified,’ meaning there were generalized complaints about a negative outcome from surgery, without specifically stating the cause (10.2% of complications). There were no reported complications of hematoma, heterotopic ossification excision, vascular injuries, femoral head or acetabular osteonecrosis, or thromboembolism on any of the three social media platforms [17, 21, 23]. In addition, posts that reported a concomitant surgery (femoral osteotomy, arthroscopy, bilateral PAO, or additional surgery) were less likely to report a complication (12.1% vs. 6.5%, *p* < 0.032153).

**Table 2:** Patient-perceived complications are shown

Perceived Complications		Instagram	Facebook	Twitter	All SM Sites (avg.)	% Total Compli- cations
**Pain**						
	Chronic Pain (> 1 week)	77 (44%)	74 (42%)	25 (14%)	176	41.8%
	Acute Pain (< 1 week)	10 (36%)	10 (36%)	8 (28%)	28	6.7%
	Nerve Pain	13 (35%)	21 (57%)	3 (8%)	37	8.8%
**Bony Complications**						
	Nonunion	0	16 (100%)	0	16	3.8%
	Fracture (Secondary)	3 (30%)	7 (70%)	0	10	2.4%
	Progression to THA	2 (100%)	0	0	2	0.5%
**Neurologic/Psychiatric**						
	Depression/Mental Health Change	3 (38%)	3 (38%)	2 (25%)	8	1.9%
	Syncope	1 (50%)	1 (50%)	0	2	0.5%
	Sleep Issues	0	0	5 (100%)	5	1.2%
	POTS	0	0	1 (100%)	1	0.2%
**Miscellaneous**						
	Anemia	0	1 (100%)	0	1	0.2%
	Infection	2 (33%)	4 (68%)	0	6	1.4%
	Constipation	0	1 (33%)	2 (68%)	3	0.7%
	Swelling	0	6 (86%)	1 (14%)	7	1.7%
	Headache	0	1 (50%)	1 (50%)	2	0.5%
	Bursitis	0	3 (100%)	0	3	0.7%
	Allergic Reaction	0	3 (100%)	0	3	0.7%
	Other Specified Complication	9 (13%)	36 (53%)	23(34%)	68	16.2%
	Non-Specified Complication	2 (4%)	22 (51%)	19 (44%)	43	10.2%
**Total Complications**		**122 (29%)**	**209 (50%)**	**90 (21%)**	**421**	**100%**

Regarding engagement, posts that included a perceived complication received a lower average number of likes and comments (14.6 likes, 6.0 comments) across all three social media platforms than posts without a complication (21.1 likes, 6.5 comments). Tweets were additionally assessed on the average number of shares per post, which decreased from 0.6 to 0.1 shares per post when posts included a complication. The results are shown in Table 3.

**Table 3:** Engagement of complication vs. non-complication posts

Social Media Platform	Complication Inclusion	Average Likes	Average Comments	Average Shares
Facebook	Complication	4.0	13.1	-
	No Complication	7.4	14.1	-
Instagram	Complication	37.7	4.5	-
	No Complication	52.8	4.9	-
Twitter	Complication	2.2	0.38	0.14
	No Complication	3.0	0.42	0.55

## Discussion

Patients use social media to learn about their conditions and share their experiences with others on their own terms. Of the 2559 posts included in this study, more than 2000 came from unique conversations related to PAO surgery in just the past two years. Patients are seeking out interaction on social media, not just for facts and statistics but for testimonials from others who have had similar experiences, and healthcare providers are taking note. Physicians can use these social media interactions to learn what patients care about when it comes to their pain and other postoperative complications, ultimately allowing them to alter their plan of care to mitigate those complications how they so choose. Utilizing social media in this way has proven to be fruitful within orthopedics. In a 2019 study, Haeberle et al. examined the social media posts of 2013 patients of hip arthroscopy to recognize trends in patient reported outcomes and complications following surgery [6]. A 2017 study found key differences between reported outcomes of patients undergoing a total hip arthroplasty and those undergoing total knee arthroplasty by analyzing patient Instagram posts [8]. These studies focused primarily on the short and intermediate postoperative timeframe of their respective procedures but did not provide analysis of patient complications in the long postoperative timeframe, a crucial consideration especially for invasive procedures with extensive long-term recovery such as a PAO. Our study is one of the largest retrospective observational studies of social media posts related to a surgical procedure, and to our knowledge, the only study focused on analyzing social media posts from patients of PAO hip surgery.

Prior studies have shown a low PAO complications profile based on traditional PROMs, however, patients’ perceived complications shared on social media may differ [17, 19]. Our study provides a data set of these perceived complications shared by patients and aims to identify other factors associated with complications. We found that most patients do not report a complication of PAO. If a complication of PAO was reported, it was most likely related to unmanageable pain. Of these, chronic pain was responsible for the majority of posts on all sites, followed by nerve pain and acute pain. This suggests that some patients may experience long-standing pain following PAO and are still searching for relief. However, very few other complications were cited at a high frequency. In particular, bony complications such as nonunion and secondary fractures were reported at a low frequency. One reason could be that given the low complication profile associated with PAO, there are fewer nonunions, secondary fractures, and other repair-requiring complications of PAO than expected. Another consideration is that patients may be unaware of the terminology referring to these more technical complications, while a complaint such as pain is understood by all. Finally, private PAO groups may also add further insight into patient perceived complications.

We additionally found that an average postoperative timeframe greater than six months existed for all posts regardless of association with complications, across all three sites. Furthermore, we found that posts including a perceived complication are more likely to be posted even later in the postoperative timeframe than posts without a complication. This suggests that patients recovering from PAO are struggling to find respite from their symptoms despite physical therapy and medications more than six months after their procedures. Additionally, we found that patients who underwent concomitant surgery with a PAO (femoral osteotomy, bilateral PAOs, arthroscopy, or other additional hip surgery) were less likely to have a post including a complication. This was unexpected as previous studies have suggested secondary surgery may increase risk of complication or compromise outcome of PAO [17, 19, 24, 25]. It is conceivable that for every additional surgery a patient undergoes, they spend more time with healthcare providers receiving education on the procedure, outcomes, and expectations. Consequently, those patients may have fewer unanswered questions about PAO that would lead them to post about their complication on social media.

We found distinctions between the complications reported in this study and prior literature. First, several past PAO outcome studies found a higher total complication rate compared to our study. In a 2018 study using traditional clinical surveying, Wells et al. noted a 43% occurrence of total complications (defined as grades 1–4 in the modified Clavien-Dindo classification system) in PAO hips [17, 26]. In another study, Biedermann et al. demonstrate a total complication rate of 78% for PAO [23]. Our study showed a much lower (13%) occurrence of complications when gathered from patient reporting in social media posts [17, 26]. Additionally, Wells et al. noted the presence of major complications (grades 3–4 of Clavien-Dindo classification) in 9% of postoperative PAO hips, which included revision PAOs, deep infections requiring incision and debridement, open reduction and internal fixation of symptomatic nonunion, and heterotopic ossification [17]. We anticipated that more major complications would be reported on social media compared to traditional measures because patients would be more motivated to search for information on how to mitigate their major complication. However, our study shows a less than 1% occurrence of posts regarding major bony complications (6% of total complications), of which only three outcomes were reported: nonunion, fracture, and progression to THA. One potential explanation, which was described by Biedermann et al., is that there was no significant difference found between the effects of major and minor complications of PAO on patient subjective health or pain reporting [23].

An additional distinction between this study and the existing literature comes from our classification of pain as its own surgical complication. We recorded a higher pain complication rate than prior studies that did not classify pain separately. Nearly 60% of the complications in our study were direct complaints of pain. Biedermann et al. found that 30% of complications were due to pain from dysesthesia and Wells et al. noted that 11% of complications were due to nerve injury, however, the proportion of patients whose primary concern was pain itself is difficult to discern [17, 23]. A mean WOMAC pain score and/or SF-36 were collected in these previous studies, effectively recording subjective patient pain levels, but these still do not clarify whether pain or the underlying complication matter most to the patient [17, 21, 23]. The nature of our retrospective observational study prevented the accurate categorization of pain by its underlying cause because patients often did not report on the source of their pain. However, while this hindered our ability to collect data on objective complications, it emphasized what mattered most to the patients themselves: their pain. The high level of patient reporting of pain on social media emphasizes the importance of informing patients and managing expectations preoperatively. Physicians inform patients of the low complication profile expected following a PAO (minor risk of nonunion, fracture, infection, DVT/PE, nerve injury) and from these interactions patients may retain a list of objective complications, but they may not know how to treat the manifestations of these complications (pain) when they arise. This reality is evident in the findings of our study, as nearly 60% of patient posts that included a complication were questions or complaints about unmanageable pain. It may prove beneficial for physicians to emphasize expected pain levels in their preoperative information sessions with patients to better inform those undergoing PAO of what levels of postoperative pain to expect, and what severity of pain warrants further clinical assessment or advanced medical intervention.

This study provides insight into the nature of social media and the different ways in which Twitter, Facebook, and Instagram are utilized. Overall, complication-related posts were found to generate less engagement on social media sites. Twitter showed a significantly lower volume of PAO posts than Instagram or Facebook and had less post engagement with fewer average likes and comments than the others. This demonstrates that Twitter users interact less frequently with other users who have experiences related to PAO surgery. Interestingly, the average timeframe of Twitter posts did not change between posts with and without a complication. Nearly 50% of complication posts were reported on Facebook, and the site showed the highest proportion of posts with a complication. Facebook showed the highest post engagement of the platforms, with the average number of comments on complication-related posts more than tripling Instagram. Furthermore, Facebook had the longest average postoperative timeframe for posts, nearly doubling that of Instagram. This emphasizes the nature of Facebook interest groups as more intimate, community-based sites for information gathering, with patients continuing to engage on the site at a much longer time after surgery. On the other hand, Twitter and Instagram were used less for asking other users about surgical complications and more for sharing recovery updates and postoperative milestones. This may provide insights for the creation of future studies utilizing social media data to represent post-surgical outcomes and complications that patients themselves feel to be important.

It is important that patients have the option to use social media platforms to share their experiences and complications after PAO surgery with others. However, healthcare providers should be wary of the potential risks of misleading or inaccurate information posted by users who are not trained healthcare professionals. Such posts could alter the perception of those who are considering a PAO surgery or even endanger or mislead patients who are actively recovering. Healthcare providers can assess the accuracy of social media posts related to their procedures and use this information to help their own patients make informed and unbiased decisions based on their health needs. In our study, some complication-related posts had replies from physicians clarifying expectations for postoperative complications, which were well-received by the original poster and other commenters. An opportunity exists for even more healthcare providers to create a social media presence and interact directly in interest groups, offering their knowledge and expertise directly to patients around the world. We anticipate an increase in physician presence on social media sites over the coming years due to the opportunity to gather patient data, answer pertinent questions, and broadcast their services. Future studies will be needed to assess the validity of patient posts before providers can use the perceived complications to alter patient care. Reaching out to patients who posted on these social media platforms to gather pertinent information and confirm statements regarding their PAO complications will help to minimize limitations.

This study has limitations. Given the retrospective nature of the study, we were unable to follow up with any posters on the three social media sites, preventing us from determining the accuracy of the statements made or confirm that they were patients of PAO. There exists potential for a subsequent study surveying posters of complications collected for this study to obtain more extensive data concerning secondary surgeries and other orthopedic complications. Second, not everyone who undergoes PAO is present on social media, and many of those who are may not share their experiences. For those who did post about their PAO, only posts which used our five selected hashtags or were in our selected interest groups were included. This may limit the generalizability of our study results, although these hashtags and groups were chosen because they were the most populated. This study has inherent selection bias, as only three social media platforms were included in analysis. Specifically, it may not address the concerns of the younger PAO audience who may be more likely to share their experiences on newer platforms such as Snapchat or TikTok. A follow up study examining posts from these additional social media platforms would be beneficial to further our understanding of the use of social media in reporting complications of PAO, although collecting data from these sites may prove challenging. This study is additionally limited by unavailable data from private Facebook PAO groups. Our goal was to be the voice of patients and utilize social media to express this. Private groups will always be available for patients who wish to not publish in non-private settings. As a result, we may have missed some complication-related posts. Finally, the average post-surgical timeframe for published posts may be misrepresented, as many of the posts were outcome reports at important milestones to the patients, such as a surgical anniversary many years postoperatively.

## Conclusions

In summary, the majority of posts related to PAO across Twitter, Facebook, and Instagram did not include a complication. Of posts with a complication, the most common was regarding unmanageable pain, followed by unspecified complications and bony complications. Complication-related posts were found to be correlated with two factors across social media sites: (1) postoperative timeframe and (2) concomitant surgery (e.g., femoral osteotomy, bilateral PAOs, arthroscopy). Posts including a complication were more likely to have a later postoperative timeframe, and less likely to originate from a patient who underwent concomitant surgery with PAO. Additionally, post engagement decreased when the post included a complication across all three platforms. Many prior studies creating complication profiles of PAO surgery describe a higher complication rate than our study, despite a lack of emphasis on unmanageable pain, though pain is reported at relatively high rates by patients on social media.

## Data Availability

The datasets used and/or analyzed during the current study are available from the corresponding author on reasonable request.

## Abbreviations

DDH: Developmental Dysplasia of the Hip
DVT: Deep Venous Thrombosis
PAO: Periacetabular Osteotomy
PE: Pulmonary Embolism
POTS: Positional Orthostatic Tachycardia Syndrome
PROMs: Patient Reported Outcome Measures
SF-36: 36 Item Short Form Survey
THA: Total Hip Arthroplasty
WOMAC: The Western Ontario and McMaster Universities Osteoarthritis Index
